# Management of Large Full-Thickness Burns Using Kerecis™ Acellular Fish Skin Graft and ReCell™ Autologous Skin Cell Suspension: A Case Report of Two Patients with Large Surface Area Burns

**DOI:** 10.7759/cureus.71101

**Published:** 2024-10-08

**Authors:** Jaclyn R Cerceo, Aldin Malkoc, Alexandra Nguyen, Amanda Daoud, David T Wong, Brandon Woodward

**Affiliations:** 1 General Surgery, Arrowhead Regional Medical Center, Colton, USA

**Keywords:** btm, full-thickness burns, kerecis, novosorb, recell

## Abstract

Novel treatments for extensive full-thickness burns revolve around fluid control, addressing systemic inflammatory derangements, and achieving early skin coverage with acceptable cosmetic and functional results. Recent advances in the management of extensive burns include fish skin xenografts, such as Kerecis Omega-3 acellular dermal substitute. Reported to be non-allergenic and antimicrobial, this Atlantic Cod skin derivative has the potential to supplement the management of patients with large surface area burns. A chart review was performed on two complex patients. Patient 1 suffered 65% partial and full-thickness burns after lighting herself on fire with gasoline, and patient 2 suffered 86% partial and full-thickness burns following a methamphetamine explosion. The patients were both treated with a multi-step process using cadaveric allografts, Kerecis acellular fish skin placement, and autologous split-thickness skin grafts (STSG). Case 2 utilized ReCell autologous skin cell suspension and Novosorb bilaminate dermal substitute (BTM^TM^) due to limited donor sites. Complete coverage and epithelization were achieved on both patients but required multiple reapplications of autograft and Kerecis. Contractures of the neck, elbows, and hand were present in Case 1. Kerecis xenografting may have an expanding role in burn management due to standalone capabilities for deep partial-thickness burns and ease of use. Further evaluation is needed to establish the most optimal timing of use and best zones of application to improve take and reduce contractures.

## Introduction

Burn management strategies have centered around stabilization of hemodynamic, respiratory, electrolyte, and metabolic parameters followed by skin grafting [1]. The current standard of care focuses on the use of either cadaveric/allogenic skin or autologous skin grafting [2,3]. Recent advances in wound regeneration include acellular fish skin dermal substitutes, including Kerecis Omega-3, a derivative of Atlantic Cod skin found in Norway. Fish skin xenografts have been used in a variety of acute and chronic wound regeneration cases including the management of burns, diabetic foot and venous stasis ulcers, and combat injuries [4]. The popularity of use has been driven by fish skin's structural similarities to human skin, which enable accelerated wound healing. Furthermore, these fish products are claimed to be hypoallergenic, antimicrobial, antiviral, and cost-effective [5,6,7]. This series focuses on two patients with extensive burns covering >65% of the total body surface area (TBSA) who underwent Kerecis cod skin xenografting in conjunction with traditional burn management strategies. Through this review, we aim to share how Kerecis application differed between these cases and how burn wound healing was impacted with each approach.

## Case presentation

Case 1

A 35-year-old female presented after self-immolation with gasoline following a domestic dispute. On initial evaluation, burns comprised 65% TBSA: second- and third-degree burns to the posterior torso, abdomen, and chest (with the majority sparing of the breasts), lower two-thirds of the face/neck, and third-degree burns to bilateral medial thighs. In addition, bilateral arms had third-degree burns circumferentially with absent radial pulses. Burn wounds were debrided at bedside upon arrival to the intensive care unit (ICU) with subsequent escharotomies of bilateral arms and chest, followed by the application of polysporin-coated xeroform to all burn areas. Substance use screening was positive for methamphetamine and alcohol. 

Throughout this hospitalization, a total of seven skin grafting procedures were performed, four of which utilized 8,650 cm^2^ in total of Kerecis Omega-3 xenograft (Table 1). The first application of Kerecis was on hospital day five. Notably, the patient experienced several failed applications necessitating re-debridement and re-application in combination with autologous skin grafting to achieve proper wound healing on par with organic re-epithelialization following burn injury. Furthermore, early admission was complicated by sepsis, with metabolic and electrolyte derangements. We noted that there was no significant improvement in terms of fluid resuscitation and there was no further improvement in pain with the use of the xenograft. After a hospital course spanning 66 days, the patient was discharged to a rehabilitation center for continued wound care and physical/occupational therapy (PT/OT). At the time of discharge, dry xeroform covered all graft sites and the patient was on room air, tolerating oral intake, and ambulating without assistance. The patient developed significant anterior neck contractures that subsequently underwent contracture release once matured. She later developed bilateral elbow, left wrist, and hand contractures.

**Table 1:** Case 1 operative details N/A: not applicable

Day	Operation performed	Graft surface area	Surgical findings	Postoperative dressing	Blood products
1	Bedside debridement of all burns and escharotomies of the bilateral arms and chest	N/A	TBSA 65%	Polysporin and xeroform (P/X)	N/A
2	Debridement of burns; cadaveric allograft applied to third-degree burns on the anterior torso, bilateral arms, medial thighs, and anterior neck	Cadaveric: 4835 cm^2^	Deep "leathery" third-degree burns	P/X	N/A
5	Tangential excision and application of Kerecis in a 2:1 mesh to third-degree burns on the posterior torso and neck	Kerecis: 2250 cm^2^, covering 3500 cm^2^	Deep third-degree burns	Polysporin and Adaptic (P/A)	N/A
13	Re-debridement and removal of allograft placed on day 2, replaced areas with the application of Kerecis	Kerecis: 5000 cm^2^	Re-debridement down to fat with thrombosed vessels	P/A	2 units pRBC
28	Tangential excision and split-thickness skin autografting (STSG) in a 2:1 mesh from a right leg donor site to bilateral arms/shoulders. Kerecis applied to bilateral thighs, right flank, and anterior torso	STSG: 2400 cm^2^, Kerecis: 700 cm^2^	N/A	STSG: P/X Kerecis: P/A	2 units pRBC
31	Debridement and replacement of Kerecis placed on day 28 to the bilateral thighs, right flank, and anterior torso	Kerecis: 700 cm^2^	Bilateral thighs with thick eschar and deep tissue infection, evidenced by necrosis, debrided with some remaining dermis; regions of the right chest, lower abdomen, and right flank demonstrated unsuccessful take/healing with Kerecis	Hydrogel/ Adaptic; Bilateral thighs: acticoat (AC)	N/A
39	Debridement of non-healing Kerecis and replacement with STSG in a 2:1 mesh taken from donor sites on the bilateral posterior thighs and left lateral/anterior thigh	STSG: 2400 cm^2^	Areas of granulated wounds with unincorporated and rejected xenograft	P/AC	N/A
62	Any non-grafted area with granulation tissue spanning > 2 cm on the posterior torso, shoulders, and left wrist was covered by STSG in a 2:1 mesh from donor sites on the left lateral buttock and thigh	STSG: 1200 cm^2^	Contractures–most notably of at-risk areas that underwent Kerecis application, especially the anterior neck spanning from the mandible to bilateral clavicles; findings instigated intraoperative Plastic Surgery consultation	P/X	N/A

Case 2

A 45-year-old female presented after an RV explosion that occurred while cooking methamphetamines. On initial evaluation, burns comprised 86% TBSA: second-degree burns to the face with scalp sparing; second- and third-degree burns to the neck, chest, upper abdomen, posterior torso, and buttocks; circumferential third-degree burns to the left upper extremity; second- and third-degree burns of the right upper extremity; and third-degree burns to the bilateral lower extremities with diminished doppler signals in all four extremities. Operative debridement and four extremity escharotomies were performed on hospital day 1. All initial burns were dressed in polysporin with xeroform.

In total, the patient underwent 12 different grafting procedures, four of which used a combined 12,050 cm^2^ of Kerecis Omega-3 xenograft (Table 2). The first application of Kerecis was on hospital day 29. Supplemental grafting agents were implemented throughout this case, including ReCell and Novosorb BTMTM. At the time of discharge, 86% of the burns were covered with autologous or Kerecis with the remaining covered with Novosorb BTMTM. Burns to the anterior torso and bilateral shoulders were covered with Novosorb BTMTM, the bilateral lower extremities had healing autologous grafts, while the right buttock, left foot, and posterior torso were healing with Kerecis. Hospitalization was complicated by multiple episodes of fungal/bacterial infections at graft sites (*Rhizopus*, *Candida*, *Klebsiella*, *Enterococcus*, and *Pseudomonas*), prompting a protracted course of broad-spectrum antimicrobial and antifungal therapy. During the hospital course, the patient endorsed suicidal ideation and was non-compliant with PT/OT recommendations. She was diagnosed with major depressive disorder and placed on an anti-depressant with moderate adherence to treatment. Despite these challenges, the patient was eventually discharged to a skilled nursing facility for continued wound care and PT/OT after approximately five and a half months.

**Table 2:** Case 2 operative details N/A: not applicable

Day	Operation performed	Graft surface area	Surgical findings	Postoperative dressing	Blood products
1	Right-sided chest tube placement, surgical debridement of extremities with four extremity escharotomies, and bedside debridement of remaining burn sites	N/A	TBSA 86%; faint Doppler signals in all extremities pre-procedure; palpable distal pulses in all extremities post-procedure	P/X Escharotomy incisions: hydrogel	N/A
2	Escharotomy of the chest	N/A	Improved chest wall compliance and ventilator synchrony post-escharotomy	P/X; Escharotomy incisions: hydrogel	N/A
3	Decompressive laparotomy and negative pressure Bogota bag placement	N/A	~3L fluid removed from abdomen; immediate increase in urine output and decrease in ventilation requirements post-laparotomy	Hospital standard Bogota with Kerlix and JP drain system entirety secured with Ioban	N/A
6	Tangential excision and cadaveric allografting to bilateral upper and lower extremities as well as repeat exploratory laparotomy with washout, partial abdominal closure, and negative pressure Bogota bag replacement	Cadaveric: 8482 cm²	Peak inspiratory pressure at the end of the case: 29	P/X; Hospital standard Bogota with Kerlix and JP drain system entirety secured with Ioban	2 units pRBCs, 2 units FFP
7	Tangential excision and cadaveric allografting of posterior torso and buttocks	Cadaveric: 3572 cm²	N/A	P/X	N/A
8	Tangential excision and cadaveric allografting of the chest and abdomen as well as repeat exploratory laparotomy with washout and full abdominal closure	Cadaveric: 1979 cm²	N/A	P/X	N/A
15	Debridement and removal of previously placed allograft to bilateral upper and lower extremities, chest, and abdomen. STSG and ReCell were applied to the chest and areas of the anterior right lower extremity taken from a donor site on the right upper thigh. Cadaveric allograft replaced on the remainder of the right lower extremity, left lower extremity, and bilateral upper extremities	Cadaveric: 9282 cm², STSG & ReCell: 2000 cm²	Several sites (left upper extremity, left thigh) with previously placed cadaveric allografting were debrided and noted to have black discoloration indicating possible fungal growth	P/X	2 units pRBC, 1 unit FFP
19	Tangential re-excision with cadaveric allografting replacement to back and buttocks	Cadaveric:3308 cm²	N/A	P/X	1 unit pRBC
29	Tangential re-excision of bilateral upper and lower extremities. Kerecis xerograft to right upper and lower extremities	Kerecis: 2450 cm^2^	Fungal growth under cadaveric grafts, most significantly on the right upper and lower extremities.	P/X	2 units pRBC
48	Tangential re-excision of the left upper and lower extremities, chest, abdomen, and bilateral feet. Kerecis placement to all debrided areas.	Kerecis: 3810 cm^2^	N/A	P/X	1 unit pRBC
56	Re-excision and Kerecis placement to right upper and lower extremities, left chest.	Kerecis: 2100cm^2^	N/A	P/X	N/A
79	Debridement followed by STSG in a 4:1 mesh with ReCell to the left torso, left upper extremity and anterior lower extremity taken from donor sites on the abdomen, bilateral groins, right lateral thigh and lateral chest. Debridement of bilateral feet, right lower extremity and left posterior extremity with application of cadaveric allograft	STSG & ReCell: 4500 cm^2^, Cadaveric:3656 cm^2 ^	Right lower extremity with areas of deep tissue necrosis.	P/X	N/A
99	Tangential excision and STSG in a 4:1 mesh with ReCell to bilateral lower extremities and left flank taken from donor sites on the abdomen and bilateral flanks. Wound vacuum placement to bilateral lower extremities and flank/abdomen	STSG & ReCell: 4000 cm^2 ^	N/A	P/X; Wound vacuum placement to bilateral lower extremities and flank/abdomen	2 units pRBC
120	Tangential excision of the back, left upper extremity and left foot. ReCell application to back taken from donor site on the back. Kerecis meshed in a 4:1 applied to back, left foot, and right buttock.	ReCell: 2000 cm^2^, Kerecis: 3.690 cm^2^	N/A	P/X	2 units pRBC
162	Debridement and application of Novasorb BTM to chest, abdomen, and bilateral shoulders.	Novasorb BTM: 1200 cm^2^	Failed grafts were noted in the chest and abdomen, some areas with minimal granulation tissue over fat. Left elbow fully granulated with other small areas of hypergranulation tissue on left upper extremity.	P/X	N/A

## Discussion

The use of fish skin as a dermal substitute in burn therapy has been growing in popularity. This is due to its cost-effectiveness, acellular nature, which reduces infection and graft rejection risk, and its ability to promote wound healing similar to in-vivo processes [4]. Studies have demonstrated that fish skin's antimicrobial properties and omega-3 fatty acids promote improved wound healing through accelerated re-epithelialization and migration of cell lineages [8,9]. In this series, patients underwent autograft, cadaveric allograft, and Kerecis Omega-3 xenografting. The majority of burns were initially covered by cadaveric allografts, which were later re-debrided and covered with either meshed STSG or Kerecis. Both patients demonstrated clinical improvement with the combination of fish skin xenografting and traditionally utilized grafting methods.

The second patient, who sustained 86% TBSA burns, was treated with Kerecis, Novosorb BTMTM, STSG, and ReCell. With limited donor sites, ReCell facilitated wound coverage over a greater surface area [10]. Kerecis demonstrated feasibility in the application, precluding the need for autologous skin grafting of superficial and deep second-degree burns. Kerecis was used in combination with ReCell, when autografts were no longer available, due to the extensive deep second- and third-degree burns on the posterior torso in Case 2. Despite the inability to use autograft with ReCell, the back was definitively managed with Kerecis with ReCell.

Kerecis use required re-debridement/replacement in both patients, but Case 2 demonstrated fewer replacement rounds in a patient with a higher initial TBSA. This may be due to staging Case 2 with allograft first, which created a better bed for Kerecis. In Case 1, the first Kerecis use was on hospital day five and was applied to zones that had not previously undergone cadaveric grafting. The next Kerecis application in Case 1 was to an area that had undergone only one previous round of cadaveric allografting. In Case 2, the first xenograft use was on hospital day 29 and was applied to areas that had undergone two previous rounds of cadaveric allografting. In both cases, Kerecis alone was effective, allowing epithelization even with the need for multiple reapplications. However, the necessity for reapplication seems to depend on the wound condition, with delayed application resulting in a higher likelihood of xenograft incorporation. At this time, more research would need to be collected before utilizing Kerecis in high-level TBSA burns. 

The functional quality of Kerecis is challenging to assess due to the limited number of cases. Notably, Case 1 experienced hypertrophic scarring, including a severe neck contracture and bilateral elbow and left wrist contracture, which were not observed in areas with autograft. While studies required by the Federal Drug Administration report no allergenic reactions to Kerecis, they do caution against its use in patients with fish allergies. Our patient did not have any documented fish allergy, so it is unclear whether this is an immunologic reaction or based on the degree of burn injury to those areas. There are only a limited number of preclinical and small cohort studies available [11]. Future larger studies are warranted to evaluate the short- and long-term effects, as well as to define the types and severities of burn injuries best suited for fish xenografts and the optimal timing of application to achieve the best results.

## Conclusions

Kerecis xenografting may have an expanding role in burn management due to stand-alone capabilities for deep partial thickness burns and ease of use. Delayed application of Kerecis often results in a higher likelihood of xenograft incorporation. Further evaluation is needed to establish the most optimal timing of use and best zones of application to improve take and reduce contractures.
